# Point-of-care ultrasound curriculum for internal medicine residents: what do you desire? A national survey

**DOI:** 10.1186/s12909-020-1949-4

**Published:** 2020-01-31

**Authors:** Tycho J. Olgers, Jan C. ter Maaten

**Affiliations:** Department of Internal Medicine, Univ Groningen, University Medical Center Groningen, Huispostcode AA41, Postbus 30.0016, 9700 RB Groningen, the Netherlands

**Keywords:** Ultrasound, Ultrasound curriculum, Internal medicine, POCUS

## Abstract

**Background:**

Point-of-care Ultrasound is a relative new diagnostic tool for internists. Since 2019, it is a mandatory skill for internal medicine residents in the Netherlands but an ultrasound curriculum still has to be developed. In this study we explored the current ultrasound training program and educational wishes from internal medicine residents.

**Methods:**

We have undertaken a national study in March 2019 using an online questionnaire. All internal medicine residents in the Netherlands were invited to respond.

**Results:**

A total of 247 from 959 (26%) residents completed the questionnaire. The majority of residents (78.6%) received less than 10 h of ultrasound training and 40% has never made an ultrasound at all. Almost all residents (92%) indicate that ultrasound is a useful skill for the internist. They report that the most useful applications are ultrasound of the inferior vena cava, kidneys, abdominal free fluid, deep vein thrombosis, heart and lungs. The main perceived barrier to perform ultrasound is the lack of availability of experts for bedside supervision.

**Conclusion:**

This study confirms the need for a national ultrasound curriculum for internal medicine residents and may contribute to the development of an ultrasound curriculum in line with residents educational needs. We should begin the curriculum with the previously mentioned applications, perceived by internal residents as most useful. Additional applications can be appended in the future. Finally it is necessary to expand the number of experts to supervise the residents.

## Background

PoCUS (Point-of-care ultrasound) is an emerging and relatively new skill for internists but little is known about the optimal content and duration of the training program to become competent [1]. In The Netherlands, the internal medicine (IM) residency training program has been updated in 2019 and this states that PoCUS is as a mandatory skill but the content of the educational program for PoCUS has yet to be developed [2]. Recently, a uniform ultrasound curriculum for internal medicine was proposed containing a blueprint for such a curriculum, in line with existing European ultrasound curricula [3–5].

This blueprint can be a starting point for a more detailed elaboration about choosing the core applications for every internist is an important issue.

It is questionable if all residents should become competent in every ultrasound application. Internal medicine is a large specialty with many subspecialties and not all applications may be useful for every subspecialty. In this way, internal medicine differs from other specialties like cardiology or intensive care medicine. Secondly, if residents are able to become competent, it is unsure if they can stay competent in each application due to limited exposure time and training opportunities within their subspecialty. These are important considerations that curriculum developers should take into account.

It is unknown if the proposed core application meet the needs of the residents. Residents are faced with increasing competency-based programs, individualized training programs and they are becoming more self-regulated learners [6]. Accounting for their wishes and needs may increase intrinsic motivation and learning process efficacy. Some studies have already investigated internal medicine resident wishes for ultrasound, but these results may not be applicable in the Netherlands and Europe due to differences in healthcare systems and local needs [7, 8].

We have undertaken a national survey to establish the needs and wishes of internal medicine residents for our national PoCUS educational program. These results can be used for further curriculum development in the Netherlands but may also apply for other European countries who are initiating ultrasound programs for IM.

## Methods

We have undertaken a cross-sectional national survey study in the Netherlands. We have invited all internal medicine residents to complete this questionnaire. The content of this questionnaire was developed by two researchers who are also national ultrasound experts in PoCUS for internal medicine. The final draft of this questionnaire was presented to the Dutch national taskforce for internal medicine ultrasound. The survey was finalized after incorporating their suggestions. The survey was distributed in March 2019 using an online survey tool (www.thesistoolspro.com). All residents for internal medicine are registered by the Dutch Internal Medicine federation (NIV). An invitation for this survey was distributed by the secretary board of the NIV. After 2 months all residents received a reminder to complete this survey. The questionnaire included demographic data, questions about current PoCUS training and practice, and questions about the perceived usefulness and wishes for PoCUS. The exact content of the survey can be found as a Additional file 1. Ethical approval was waived by our local medical ethics committee.

The Dutch residency program for internists consist of 4 years general internal medicine with several rotations. After 4 years they start their fellowship within a subspecialty or extended rotations for a multiple profile. Residents for other specialties (for example cardiology, respiratory medicine, gastro-enterology) follow 2 years of common trunk general internal medicine. Results are displayed as frequencies.

## Results

A total of 247 of 959 residents completed this survey yielding a response rate of 26%. The demographics of the respondents are shown in Table 1.

**Table 1:** Demographics of residents

Demographics	N 247 (%)
Age (years)	
26–30	93 (37.7)
31–35	130 (52.6)
36–40	23 (9.3)
41–45	1 (0.4)
Sexe	
Male	83 (33.6)
Female	164 (66.4)
Residency (year)	
1	33 (13.4)
2	48 (19.4)
3	43 (17.4)
4	48 (19.4)
5	41 (16.6)
6	33 (13.4)
Other	1 (0.4)
Subspecialty	
General	41 (16.6)
Vascular	6 (2.4)
Geriatrics	19 (7.7)
Endocrinology	15 (6.1)
Nephrology	13 (5.3)
Hematology	9 (3.6)
Oncology	22 (8.9)
Acute medicine	14 (5.7)
Infectious disease	11 (4.5)
Intensive care	14 (5.7)
Common trunk internal medicine	76 (30.8)
Common trunk other	4 (1.6)
Other	3 (1.2)
Current type of hospital	
Academic	129 (52.2)
Non-academic top clinical teaching hospital	92 (37.2)
Non-academic normal teaching hospital	26 (10.5)
Ultrasound machine available	
Yes, handheld	9 (3.6)
Yes, mobile	136 (55.1)
Yes, both	7 (2.8)
None	93 (37.7)
Missing	2 (0.8)
PoCUS educational sessions	
Yes	92 (37.2)
No	155 (62.8)

This shows a heterogeneous distribution of subspecialty and year of residency.

The first part of the questionnaire concerned questions about current use of and education in PoCUS. The IM residents report that PoCUS is used by internists in the minority of hospitals (32.8%) in contrast to their reported use of PoCUS by emergency physicians (66.8%). They state that PoCUS educational sessions are only available in the minority of hospitals (37.2%). More than half (55.9%) of residents did not have any previous ultrasound training (Table 2) and another 22.7% had less than 10 h of ultrasound training.

**Table 2:** Ultrasound training hours received during current residency

Course hours followed	N (%)
0	138 (55.9)
1–10	56 (22.7)
11–20	28 (11.3)
21–30	11 (4.5)
31–40	4 (1.6)
41–50	5 (2.0)
> 50	5 (2.0)

**Fig. 1:**
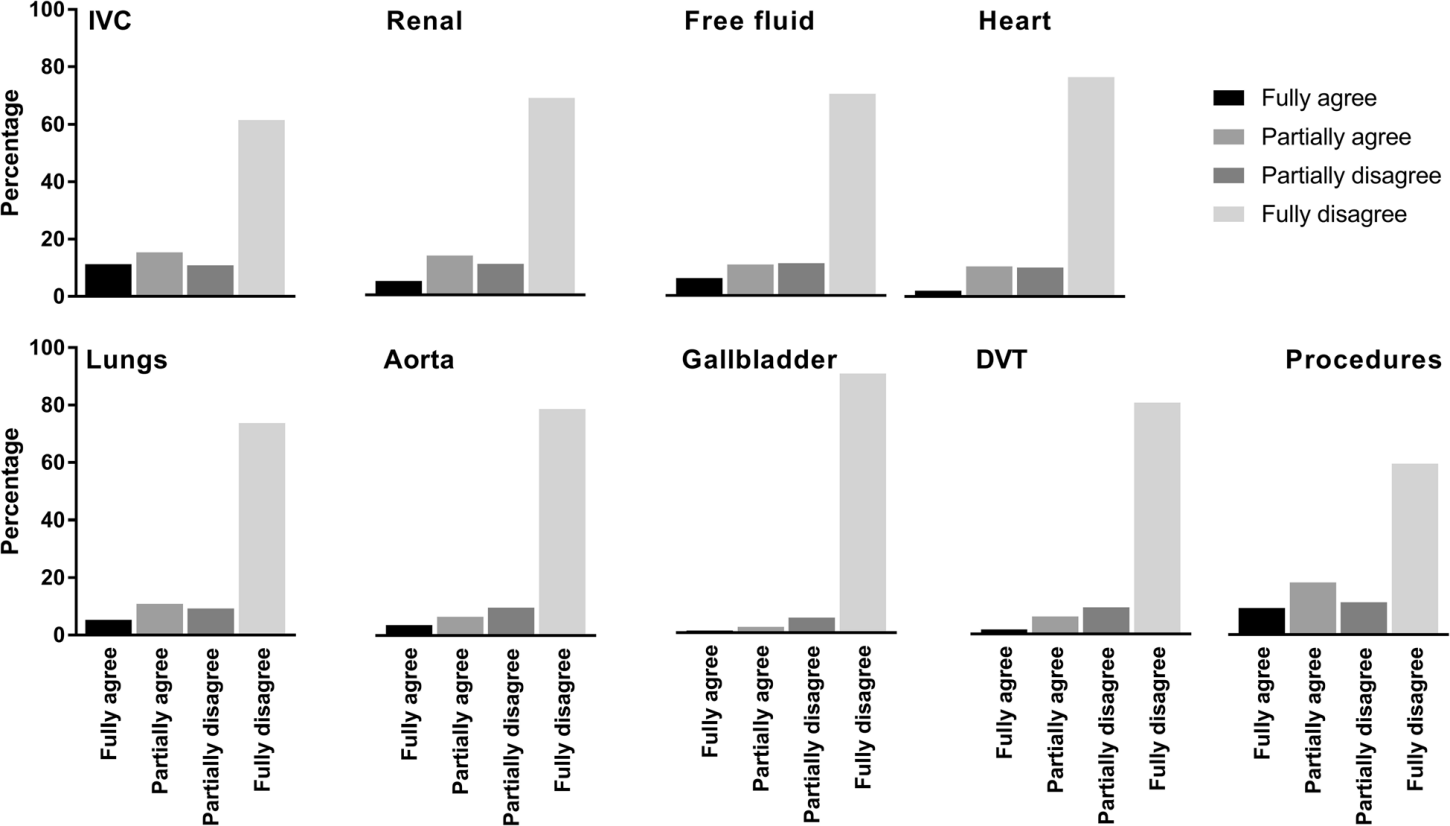
Perceived competence of residents for ultrasound applications

A significant part of residents (40%) has never made any ultrasound study at all. If residents have performed ultrasounds themselves, the most common application was the inferior vena cava (IVC) (*N* = 90 (36.4%)). Some residents feel competent for themselves for a few applications but most residents feel completely unqualified (Fig. 1).

The second part of the questionnaire investigated the perceived usefulness and future expectations. Respondents indicated that the most useful diagnostic core applications are IVC, renal and abdominal free fluid, followed by deep vein thrombosis, heart and lungs (Table 3). Aorta and gallbladder are considered least useful. Strikingly, 20 residents (8.1%) think that not a single indication is useful for every internist, the reason for this was not registered. Finally, other useful indications mentioned were thyroid ultrasound and determining hepatosplenomegaly.

**Table 3:** Most useful Core applications indicated by respondents

Core application	Yes N (%)	No N (%)
IVC	197 (79.8)	50 (20,2)
Renal	188 (76,1)	59 (23,9)
Abdominal Free Fluid	156 (63,2)	91 (36,8)
Procedures	148 (59,9)	99 (40,1)
Lungs	122 (49,4)	125 (50,6)
DVT	97 (39,3)	150 (60,7)
Heart	79 (32)	168 (68)
Aorta	42 (17)	205 (83)
Gallbladder	30 (12,1)	217 (87,9)
None	20 (8,1)	227 (91,9)
Other	4 (1,6)	243 (98,4)

Most residents (77.3%) are convinced they will use PoCUS within their own working environment

The final question was about perceived barriers. The overwhelming majority (96.8%) experiences at least one limitation for performing PoCUS (Table 4)

**Table 4:** Perceived barriers for PoCUS use

Limitations for ultrasound	N (%)
Insufficient experts available	150 (60,7)
Insufficient supervised practice time	142 (57,5)
Insufficient knowledge of PoCUS	140 (56,7)
Insufficient practice time	126 (51)
Insufficient training available	103 (41,7)
No ultrasound machine	82 (33,2)
No national guideline from NIV^a^	53 (21,5)
Resistence from other specialties	51 (20,6)
Other	27 (10,9)
No limitations	8 (3,2)

^a^*NIV* Nederlandse Internisten Vereniging (Dutch Internal Medicine Federation), *PoCUS* Point-of-care ultrasound

The main perceived barriers are insufficient experts available for supervision, insufficient knowledge and skills about PoCUS and lack of time for practicing ultrasound. Other barriers were expecting limited exposure time to PoCUS and therefore doubting the usefulness of PoCUS for themselves. Finally, issues with financing ultrasound courses was mentioned several times.

## Discussion

Our study shows that PoCUS education and experience is very limited for IM medicine residents in the Netherlands but very desired. Residents are almost unanimous that PoCUS will provide better and faster patient care and most of them believe they will use PoCUS within 5 years. Residents seem to have clear ideas what they think is important for their education in PoCUS and this is in line with previous studies [7, 8]. Curriculum developers should allow for their wishes to optimize the PoCUS educational program.

Although many ultrasound curricula already exist, some of which are very extensive, it is questionable if every country can fully adopt these curricula due to local and national differences in healthcare structure [9, 10]. Also, there is no consensus at this moment on the content of ultrasound curricula specifically for internal medicine [11, 12]. It is necessary to make choices in core applications because IM is a large specialty with many subspecialties. To become, and stay competent in PoCUS, enough training hours and patient encounters are mandatory but this may not be achievable for every IM resident. At this moment, it is not known how many training hours or performed exams are needed to become competent for each application. Some studies show that limited exposure time is sufficient to master IVC ultrasound [13]. The American college of radiology demands for non-radiology physicians performing ultrasound to follow at least 200 h category 1 continuous medical education in the subspecialty where ultrasound reading occurs, and supervision and/or performance, interpretation, and reporting of 500 cases relative to each subspecialty area interpreted (e.g., pelvic, obstetrical, thyroid, vascular) during the past 36 months in a supervised situation [14]. For IM residents the optimal training hours and number of exams performed to become competent has to become clear but using the entrustable professional activities (EPA) system might assist [3]. This system defines competence and need for supervision on five different levels and is based on the observed ultrasound performance instead of a fixed number of ultrasound studies.

We have to design ultrasound curricula with a basic set of core applications useful for every IM resident, that can be extended with more specialized application depending on residency year and subspecialty, and are in line with local and national healthcare wishes and regulations. Ultrasound introductory courses should focus on these core applications and limit the total number of applications.

We have shown that residents have clear ideas how educators should construct the ultrasound curriculum specifically the most useful core applications for IM. According to IM residents in the Netherlands, IVC, renal and abdominal free fluid, should be the core diagnostic ultrasound applications, supplemented by deep vein thrombosis, heart and lung ultrasound. Ultrasound curricula can be designed in a way that all residents become competent for these applications. Additional applications can be learned at later stages of their residency program and will be determined by residents own wishes, subspecialty and regional healthcare structure.

Finally we have to increase the number of ultrasound experts who can supervise the residents. At this moment there is insufficient time to practice, especially practice time together with an expert.

### Limitations

Our study may be limited by a response rate of 26%, although this is a reasonable response rate for survey studies (mean web-based data collection response rate 27.6%) [15]. Nonetheless, it is possible that a selection bias exist with responders being more enthusiastic about ultrasound with different ideas than non-responders. We did not contact a subgroup of non-responders. Also, we did not test our survey in a small group in advance so misinterpretation of questions cannot be excluded. Finally, we did not have open questions, so other important issues regarding ultrasound education may be missed.

## Conclusion

We have shown that IVC, renal, abdominal free fluid, deep vein thrombosis, cardiac and lung ultrasound are perceived as most useful core applications for IM residents by IM residents. Ultrasound curricula need to take these wishes into account. These applications should be the basic core applications for ultrasound courses and the national ultrasound curriculum. They can be extended by several other more specialized applications depending on year of residency, subspecialty and regional healthcare structure with additional training and courses.

## Supplementary information

**Additional file 1.** Survey POCUS Internal medicine.

## Data Availability

Supporting data can be requested from the corresponding author by email (t.j.olgers@umcg.nl).

## Abbreviations

EPA: Entrustable professional activities
IM: Internal medicine
IVC: Inferior vena cava
PoCUS: Point-of-care ultrasound
