# Supplementary material for: Point-of-care ultrasound curriculum for internal medicine residents: what do you desire? A national survey
Source: BMC Med Educ. 2020 Jan 31;20:30. doi: 10.1186/s12909-020-1949-4 (PMC6995217; doi:10.1186/s12909-020-1949-4)
Supplement: Supplementary file 1 — Additional file 1. Survey POCUS Internal medicine. [file 12909_2020_1949_MOESM1_ESM.docx]

**Survey POCUS Internal medicine**

POCUS (Point-of-care Ultrasound POCUS) is a commonly used diagnostic tool in several specialties. It is also used in emergency medicine and acute internal medicine for several years now. Until now, it is unclear for internal medicine residents how they can become competent in POCUS, which possibilities and opportunities are available, which limitations are encountered and what their own needs are. We would like to ask you to complete this survey to elucidate these issues. We hope to optimize the national internal medicine ultrasound curriculum with these results.

We would appreciate your participation. The results will be analyzed anonymously. Your participation is completely voluntary. The survey consists of 20 questions and will take you about 5 minutes to complete.

1. **What is your age?**

A 20-25 B 26-30 C 31-35 D 36-40 E 41-45 F 46-50 G >50

1. **What is your gender?**

A male B female

1. **In which year of residency are you?**

A 1 B 2 C 3 D 4 E 5 F 6 G other

1. **What is your specialty?**

A general internal B vascular C geriatrics D endocrinology

E nephrology F hematology G oncology H acute medicine I infectious disease

J common trunk (internal medicine) K common trunk other (cardiology, GE, pulmonary) L other

1. **What is your current workplace?**

A academic B large community hospital C small community hospital

1. **To what degree do you agree or disagree with the following statement? POCUS is being performed frequently by internists in our hospital.**

Partly agree means: POCUS is being used but not frequently

Partly disagree means: POCUS is being used occasionally

A totally agree B partly agree C partly disagree D totally disagree

1. **To what degree do you agree or disagree with the following statement? POCUS is being performed frequently by emergency physicians in our hospital.**

Partly agree means: POCUS is being used but not frequently

Partly disagree means: POCUS is being used occasionally

A totally agree B partly agree C partly disagree D totally disagree

1. **Do you have access to an ultrasound machine?**

A yes, handheld B yes, mobile machine (laptop or machine on wheels C yes, both D none

1. **To what degree do you agree or disagree with the following statement? POCUS is a diagnostic modality for physicians that contributes to faster and better diagnosis for several conditions in internal medicine.**

A totally agree B partly agree C partly disagree D totally disagree

1. **To what degree do you agree or disagree with the following statement? POCUS is useful for me personally in acute patient care.**

A totally agree B partly agree C partly disagree D totally disagree

1. **Which POCUS applications do you think are useful for EVERY internist? (multiple answers possible)**

A none B heart C lung D vena cava E renal and urinary tract F free fluid G aorta H gallbladder I deep vein thrombosis J procedures (IV access/paracenteses) K other

1. **Which POCUS applications do you think are useful for your OWN subspecialty? (multiple answers possible)**

A none B heart C lung D vena cava E renal and urinary tract F free fluid G aorta H gallbladder I deep vein thrombosis J procedures (IV access/paracenteses) K other

1. **Are ultrasound training sessions for internists being held in your hospital?**

A yes B no

1. **How many hours of ultrasound courses did you attend? Please specify which courses).**

A 0 B 1-10 C 11-20 D 21 – 30 E 31 – 40 F 41-50 G > 50 hours

1. **How many ultrasound have you made yourself?**

A 0 B 1-20 C 21-60 D 61-100 E 101-200 F >200, estimation….

1. **And for which applications? (multiple answers possible)?**

A not applicable B heart C lung D vena cava E renal and urinary tract F free fluid G aorta H gallbladder I deep vein thrombosis J procedures (iv access/paracenteses) K other

1. **To what degree do you agree or disagree with the following statement? I feel qualified to perform POCUS for my own working situation.**

A totally agree B partly agree C partly disagree D totally disagree

1. **To what degree do you agree or disagree with the following statement? I feel qualified to perform POCUS without supervision:**

Totally agree partly agrees partly disagrees totally disagree

vena cava

renal

free fluid

heart

lungs

aorta

gallbladder

deep veen thrombosis

procedural (IV access/paracentesis)

1. **What do you feel are the biggest limitations or barriers to learn or perform POCUS at this moment? (multiple answers possible)**

A Insufficient knowledge of POCUS

B Insufficient training available

C Insufficient experts/supervisors available for support

D No US machine available

E Insufficient practice time

F Insufficient practice time with supervisor

G Possible resistance from other specialties (for example radiology, cardiology)

H No guideline available from Dutch Internal Medicine foundation or others

I Other, namely……

J No barriers or limitations

1. **To what degree do you agree or disagree with the following statement? It is very likely that i will use POCUS within 5 years for my own specialty.**

A totally agree B partly agree C partly disagree D totally disagree E I already use POCUS
